# Falsified Medicines—Bridging the Gap between Business and Public Health

**DOI:** 10.3390/pharmacy4020016

**Published:** 2016-03-28

**Authors:** Rasmus Borup, Janine Traulsen

**Affiliations:** 1 Department of Pharmacy, University of Copenhagen, Universitetsparken 2, Copenhagen 2100, Denmark; Janine.Traulsen@sund.ku.dk; 2 Division of Pharmacoepidemiology & Clinical Pharmacology, Utrecht University, Universiteitsweg 99, Utrecht 3584CG, The Netherlands

**Keywords:** falsified medicines directive, pharmaceutical policy, European commission, multiple streams framework

## Abstract

The pharmaceutical industry is one of the most regulated industries in the world. While legislation is necessary to protect patients, too much legislation is said to hamper innovation and increase medicine prices. Using qualitative methods such as interviews and document analysis, we investigated the role of private stakeholders in the EU policymakers’ decision to initiate legislation to combat falsified medicines in 2008. Our results show that the pharmaceutical industry, brand owners in particular, were strong proponents of legislation to combat falsified medicines. Their support was not fueled by fear that falsified medicines would harm patients or their own business, but rather because legislative action in this area would advance policies that benefit their businesses objectives. The brand owners framed the issue to policymakers as best to support their business objectives. In general, supply chain actors lobbied for stricter requirements in order to challenge competitors. In the end, the Falsified Medicines Directive may have suffered from company influence not by addressing the primary problem of falsified medicines, but rather by creating additional legislation that benefits the supply chain actors.

## 1. Introduction

The importance of pharmaceutical legislation cannot be overestimated. Much pharmaceutical legislation is a consequence of tragedies such as that of Thalidomide 50 years ago and is generally considered to be a necessary response to under-regulation [1]. However, legislation has often been accused of setting too high standards resulting in over-regulation, thereby interfering with businesses and hampering innovation [2]. Ultimately, legislation should effectively manage the risks to patients, while simultaneously promoting access to affordable medicines and stimulating innovation in new and better products.

In social pharmacy, we explore the social constructs that influence the application of pharmaceutical science. Legislation is one such social construct. The pharmaceutical industry is often said to be one of the most heavily regulated industries [3,4], and legislation is therefore an obvious area for research. Arguably, pharmaceutical legislation should promote the application of good pharmaceutical science, thereby benefiting public health in general and patients in particular. That being said, pharmaceutical legislation cannot blindly ignore the business environment in which most medicines are developed, manufactured and marketed, and which contributes to society by creating jobs, paying taxes as well as aiding public health. Consequently, policymakers are tasked with the difficult exercise of balancing business and public health objectives when devising new legislation.

Some researchers suggest that policymakers focus too much on deregulation [5], most often arguing that policymakers are biased in favor of business rather than patients [6,7]. Other researchers argue that legislation hampers innovation, and that regulators and policymakers are overly risk averse, in effect slowing down development of medicines and causing price increases [8,9,10]. Researchers who study how pharmaceutical legislation is created usually study the subject on a macro level. We wish to illuminate the apparent gap between these two viewpoints by investigating the making of pharmaceutical legislation on a micro level.

In this article, we wish to present how pharmaceutical legislation changes. What determines which areas require change: business or public health interests? To study the question, we used a case study approach by examining the Falsified Medicines Directive (FMD) [11]. The FMD was published by the European Union (EU) in 2011 and, when finally implemented in 2019, will drastically change the way medicines are distributed from manufacturer to pharmacy [12]. The FMD calls for attaching safety features to medicine packs, as well as tracking each individual pack of medicines, which will require companies to invest hundreds of millions of Euros in technical instruments [13]. As a result, the FMD has been criticized for placing too large a burden on companies [14,15].

Although EU member states have maintained their autonomy in some areas, much pharmaceutical legislation originates from Brussels [16]. As a result, today, the role of national health authorities is increasingly to enforce legislation rather than create it [17]. In Brussels, the decision to legislate often lies with the European Commission [18]. The Commission has the role of proposing legislation. The Council and the Parliament in turn reject, change, or accept these proposals for legislation [19,20].

In this article, we wish to elucidate how the FMD came to existence. We therefore ask: what made policymakers prioritize the problem of falsified medicines? We place particular emphasis on the role of private stakeholders in our investigation.

## 2. Methods

In our study, we focused on the initial phase of the policy process: the making of the proposal to the FMD by the European Commission. In order to study how private stakeholders influenced falsified medicines reaching the legislative agenda of the European Commission in 2008, we conducted both qualitative interviews and document analysis.

### 2.1. Interviews

The FMD affects companies in the regulated supply chain by requiring them to change their procedures for handling medicines and to invest in new equipment. We assumed that these affected companies had followed the developments of the FMD closely and were the best sources to explain their own activities as well as that of other stakeholders. We identified different categories of companies that make up the regulated supply chain. The categories identified were active pharmaceutical ingredient manufacturers (API manufacturers), pharmaceutical brand owners (also known as innovative manufacturers), generic manufacturers, parallel traders, wholesalers, and pharmacies. Companies supplying medicines outside the regulated supply chain (often illegally) or otherwise unaffected by the FMD (e.g., doctors and retail shops selling medicines) were not considered for interviews. However, as the main victims of falsified medicines, consumers were also considered for interviews.

Through an initial review of the media coverage, we identified the main interest groups representing the affected categories of companies. In an attempt to raise the validity of the information given, we identified both Brussels based and nationally based (Dutch) organizations, representing the same categories of companies. A total of 17 interest groups were approached by phone or email. Individuals from 12 organizations responded and agreed to be interviewed regarding the process of drafting the FMD. The first author conducted the 12 interviews from January to March 2015. Seven interviewees were employed by European interest groups based in Brussels, while five interviewees were employed by national interest groups based in the Netherlands.

Interviews were semi-structured and followed an interview guide consisting of three main themes: the (framing of the) problem of falsified medicines, the identification of events that made policymakers prioritize legislation to combat falsified medicines, and the process of creating the FMD (including the role of the interviewees).

We also approached individuals, current and former employees of the European Commission, but none of them wished to be interviewed. All interviewees were promised anonymity.

### 2.2. Documents

To supplement and confirm information given in interviews, documents relating to the FMD were gathered through European Union websites, interest group websites, access to information requests to the European Commission, and media websites (mainly “Scrip Regulatory Affairs” and “European Voice”). Documents referring to EU pharmaceutical legislation against fake, falsified or counterfeit medicines in the period from 2004 to 2008 were included in the analysis. During or after interviews, some interviewees also supplied documents not immediately available to the public.

Documents used were press releases, hearings, responses to hearings, position papers, reports and assessments, presentations, meeting summaries, draft legislative proposals, summaries of legislative proposals, responses to inter-service consultation, and newspaper articles.

### 2.3. Analysis

Interviews were transcribed verbatim by the first author. Interview transcripts were analyzed through inductive qualitative content analysis, whereby themes were developed from the interview transcripts through repeated examination and comparison. Key issues transcending multiple transcripts were highlighted by the first author. A rough timeline for events related to these themes was created. Events identified through interviews were verified either through other interviews or by comparison to documents.

Several different theories were discussed with regard to making sense of the transcripts. It was decided that Kingdon's multiple streams framework provided the best means for understanding the data and it was therefore used when analyzing the data [21].

Kingdon proposed that three independent streams determine if an issue reaches the political agenda:

• The problem stream
• The policy stream
• The politics stream.

The problem stream represents the issue that is brought to the attention of policymakers. Different mechanisms influence whether or not an issue will attract policymakers’ attention: indicators illuminating the issue such as new data or reports; focusing events such as disasters; and other feedback channels such as media and public opinion.

The policy stream entails the existing ideas to solve or handle the problem. These ideas have promoters, often in the form of lobbyists, who try to convince policymakers that their ideas are the most suitable. Kingdon refers to these promoters as “policy entrepreneurs” and explains that they “lie in wait in and around government with their solutions at hand, waiting for problems to float by to which they can attach their solutions, waiting for a development in the political stream they can use to their advantage” [21] (pp. 165–166).

The politics stream contains factors that affect the political environment. This stream is affected by change in government or personnel, public opinion, and interest groups, among others.

These three streams must converge in order to get the political attention to use an available proposal to solve an existing problem.

## 3. Results

Stakeholders had different approaches to influencing the FMD: Brand owners and API manufacturers both lobbied policymakers to initiate legislation to combat falsified medicines. Wholesalers, pharmacies and consumers did not lobby for legislation but were active in trying to shape the adopted solutions. Parallel importers and generic manufacturers, although sympathetic to efforts to combat falsified medicines, actively opposed the measures initially proposed by the FMD in an attempt to exempt their own products from the scope of the FMD.

The first proposal to the FMD addressed the threat of falsified medicines reaching European consumers through the regulated supply chain. The proposal followed a public consultation in March 2008 where interested parties were invited to comment on some key ideas proposed by the European Commission. All interviewees responded to the public consultation by recommending specific legislative measures. The FMD proposal was published in December 2008 and states that:

“There is an alarming increase in the EU of medicinal products which are falsified in relation to their identity, history or source. […] They pose a major threat to European patients and European industry and there are strong concerns in the public and amongst policymakers about the steady increase of these products detected in the EU in the last years.” [22]

### 3.1. Problem Stream

Although all interviewees officially offered their support to combat falsified medicines, only API manufacturers and brand owners had actively lobbied to place the issue on the legislative agenda:

“We were on this issue more on the push side […]. Basically we proactively put the issue of anti-counterfeiting on the table.” (Representative of brand owners’ interest group)

One way that brand owners were trying to convince policymakers to pay attention to falsified medicines was by gathering evidence themselves. Since 2003, they had collected information to support their claim that parallel trade was unsafe. This had resulted in a file documenting 1300 instances where brand owners found the packaging material of parallel traded medicines to be visibly substandard. None of the instances was actual evidence of falsified medicines, but the file helped question the safety of parallel trade:

“We were in some way linking parallel trade with counterfeiting. But we were more making the issue that parallel trade was potential entry points for substandard medicines into the supply chain. […] We went not in the press with all these negative examples about parallel traded products at the time. But we did collect this and it was very important evidence in order to get the issue really on the top of the Commission’s agenda.” (Representative of brand owners’ interest group)

API manufacturers had little evidence of falsified medicines in the supply chain to support their argument. Their strategy to convince policymakers of the need for legislative action highlighted the future risk of doing nothing:

“Sometimes I would even personalize it. I said ‘How about you? I am sure you are taking medicines. Have you any idea where it is coming from? Or what quality it is?’ And they look at you strangely. I said 'And what about your... Have you got any grandchildren? Or you got any elderly parents? Yes?” (Representative of API manufacturers’ interest group)

Although most interviewees believed that the problem of falsified medicines existed primarily on the internet, and not in the regulated supply chain as targeted by the FMD proposal, only parallel importers and generic manufacturers promoted this view:

“It was quite clear that 95%, 96% if not 99% of all the falsifications are in the so-called internet circuit, rather than in the normal chain of logistics to deliver the goods, the medicines, to patients. So we argued in those days that by introducing this system we did not really approach or tackle the root cause of the problem, which is the internet.” (Representative of generic manufacturers’ interest group)

Several interviewees decided that opposing legislation to combat falsified medicines would weaken their ability to influence the directive later on. They therefore supported the FMD, although they did not agree with the framing of the issue. As a representative of a pharmacy interest group expressed it:

“It’s quite difficult for us to oppose strong patient safety measures inside our core business. […] We took a decision not to kill it or try to kill it, which we would never have succeeded in doing anyway. We played along with it and we tried in the subsequent seven years to shape it, which we have done reasonably successful[ly].” (Representative of pharmacy’s interest group)

### 3.2. Policy Stream

In interviews, stakeholders explained their efforts to promote policy proposals that would benefit the interests they represented. In essence, they acted as policy entrepreneurs by trying to attach policies to the perceived problem. Often these policies were preconceived ideas not directly designed for the problem at hand:

“So most of the measures that you find in the Falsified Medicines Directive were... or some of the provisions were on [our] shopping list. But it was not articulated the way the directive did at the end.” (Representative of brand owners’ interest group)

Wholesalers explained that they tried to use the FMD to make it more difficult to obtain and maintain a license to operate, thereby challenging low-resource wholesalers not able to comply with increased requirements:

“So we were even proposing to increase the rules—to make the requirements and the criteria stricter. […] But then you get into the competitive area […] where we as association have to be very careful.” (Representative of wholesalers’ interest group)

European API manufacturers lobbied for a proposal that would protect their business by elevating requirements for competitive APIs imported from outside the EU. The API manufacturers explained during interviews that prior to the FMD, they had failed to convince policymakers of the necessity of their proposal:

“I am thinking ‘Hang on a minute. Are you European? Are you supposed to be helping European industry? […] Why are you not wanting to help people who are operating in Europe, who have been made to suffer competitively, because of the lack of enforcement of legislation? Why would you not want to help them improve their competitiveness?’ Let's just leave it at that. And eventually [they] did. And every bit of—I think it was our second or third position paper—[they] put into the pot, and then [they] told us that an impact assessment was being done on something that was going to be called the Falsified Medicines Directive or whatever the euphemism was at the time, and it would all be taken into consideration.” (Representative of API manufacturers’ interest group)

The interest group representing consumers tried to persuade policymakers to include policies regulating internet sale in the FMD but did not otherwise lobby for changes to the existing legislation:

“Our response to that consultation was […] shorter and less detailed, because we had less to say, in the sense that counterfeit is illegal by definition.” (Representative of consumers’ interest group)

Brand owners had two major policy proposals that they promoted as solutions to the problem of falsified medicines: requiring safety features on medicine packs, and establishing a harmonized coding system for all European medicines.

The proposal to require safety features on medicine packs came with a condition that proved detrimental to parallel traders, whose business case relies on the right to repackage medicines:

“When you repackage, by necessity you must remove these safety features. And the directive says you can remove the safety features on condition that you do that with the approval of the brand owner. Now in our business, the brand owner would certainly never approve.” (Representative of parallel trade’s interest group)

Brand owners had wanted to limit parallel trade for more than 20 years, and they saw an opportunity to use the FMD to try to raise the legislative requirements for parallel traders:

“It’s clear that we wanted also to act in some way on parallel trade. [...] and I can share with you the response at that time we did to one of the public consultations. I think that the only sentence was that ‘the Commission is applying two sets of standards: one for (parallel traders) and one for (brand owners)’. And this is what we have been demonstrating, why we wanted a ban on repackaging.” (Representative of brand owners’ interest group)

Brand owners explained in interviews that the second main policy proposal, a harmonized coding system for all European medicines, had been on their shopping list since the 1990s. They initially proposed the idea to the European Commission in 1994, but the Commission decided not to take it further, realizing that member state authorities would not want to change their existing coding systems for no obvious benefits. Around 2006, brand owners tried to persuade other private stakeholders to form a coalition of stakeholders that were positive towards a harmonized coding system, in order to pressure the European Commission to take legislative action. However, this attempt also failed, as explained by a representative of pharmacies:

Interviewee: “[Brand owners] initially approached us way back in 2006, 2007 about setting up a system. And we went to a couple of meetings with [brand owners] and then we withdrew.”

Interviewer: “For what reason?”

Interviewee: “Because it became clear at the time that what [brand owners] was interested in was the data. Dispensing data. Which was a big issue [...]. So we thought 'Well, this has no interest for us”. […]

Interviewer: “They used counterfeit medicines as a sales point here?”

Interviewee: “Well, I can’t put words into their mouths, but currently the industry spends a lot of money on getting data, sales data for products. And there was undoubtedly a view held by some people—this was never said publicly—that the […] system could be used to harvest that data. Which is true: it could be used to harvest that. […] Well, I mean pharmacists already do sell data. But what they do is, they sell sample data. Which isn't as comprehensive as it could be. And they sell it for little. So there is a data business to be had. Although no one... It's a bit of a taboo to talk about it.” (Representative of pharmacy’s interest group)

The idea of a harmonized coding system was eventually adopted by policymakers via the FMD as a way to keep falsified medicines out of the regulated supply chain. Brand owners explained in interviews that the system had many possible versatile applications, mentioning amongst others access to dispensing data, the ability to easily change the price of medicines, and to replace printed patient information leaflets with digital versions:

“So for me it is one of the best projects that we have been working on at level of this organization over the last two decades. It's something […] that people still underestimate the potential impact. Just because we want to use the system beyond […] its pure objectives.” (Representative of brand owners’ interest group)

### 3.3. Politics Stream

The brand owners’ proposal to harmonize coding of medicines was adopted without much controversy. However, not surprisingly, the proposal to essentially ban parallel trade was met with fierce opposition from parallel traders:

“We lobbied against Verheugen’s initial proposal, and we [asked our members] to write letters to their national commissioner. We provided them of course with templates through our agency. We asked for meetings. We were granted meetings. I joined one in the cabinet of the Portuguese president at the time. One with the French commissioner’s cabinet. The Italian. And also one of the Baltic states. And Mrs. Wallström from Sweden. And there was an Irish commissioner as well. And a couple of commission or cabinet experts, who understood the matter and could clearly see that this was not legal […]. They were probably a little more liberal minded also given their commissioner’s position. They were quite supportive inside the Commission to help us spread our message. In the end, we were told that 17 commissioners voted against Mr Verheugen’s proposal when he brought it into the Commission. […] We as parallel distributors had won an important step in the fight against a directive [that] was biased and would have clearly stopped our business.” (Representative of parallel trade’s interest group)

The European Commission was divided internally with regard to which measures were justified in the fight against falsified medicines. The department of Enterprise (DG ENTR), which was responsible for pharmaceutical legislation, pushed for a ban on repackaging, whereas the department of Competition (DG COMP) opposed such drastic measures without additional knowledge of the problem:

“With respect to counterfeit products traded inside the Community DG ENTR essentially seems to rely on three instances where these products entered into the legal supply chain. […] We would still believe that further fact finding would be very useful and indeed necessary. Otherwise, it remains difficult to assess the gravity of our common concern on counterfeit products. It is also difficult to identify the appropriate measures to be taken.” (Document from inter-service consultation of the European Commission sent by DG COMP to DG ENTR)

DG ENTR did not succeed in convincing all Commission departments that a ban on repackaging was necessary, and parallel traders were allowed to continue to repack medicines, provided that they re-establish the safety features.

## 4. Discussion

The FMD was formally undertaken to protect European patients and European companies from the threat of falsified medicines. However, our results indicate that the directive was not made at the request of patients or consumers. As a representative of consumers explained:

Interviewer: “Did you before 2008 lobby for counterfeit medicines to come on the agenda?”

Interviewee: “No, I don’t think so.”

Interviewer: “Why not?”

Interviewee: “No, I... I think it was... Yeah, it was not something that was felt strongly about from our members.” (Representative of consumers’ interest group)

We observed early on in our analysis that the brand owners were the most adamant proponents of the FMD among our interviewees. However, brand owners themselves did not perceive falsified medicines as something that threatened their business. The importance of combating falsified medicines was assessed by an interest group representing more than 35 brand owners:

“I know that only two companies will put anti-counterfeiting among their top five priorities. Two companies!” (Representative of brand owners’ interest group)

Although falsified medicines were not a priority for companies or consumers, brand owners and API manufacturers lobbied to position falsified medicines in the regulated supply chain as a problem in need of a legislative solution.

To an outsider, lobbying to promote an increase in requirements rather than deregulation may seem counterintuitive—particularly when the topic is of low priority. However, by lobbying for stricter requirements, companies aimed to improve their business position, primarily by challenging competitors whose standards were considered to be lower than the proponents of legislation.

Similar rent-seeking behavior has also been described in case studies focused on environmental and consumer protection, where industry uses public health issues to lobby for policies that will benefit their business. For example, large companies may lobby for stricter requirements that smaller companies cannot afford to implement [23].

API manufacturers, who lobbied for legislative change, summarized the effectiveness of their efforts:

“So now you’ve got lots and lots of more legislation, some of which is still being awaited to come to fruition. Yeah... In some ways it’s very sad. In other ways, we were partly successful. So... I guess that's the bottom line for me.” (Representative of API manufacturers’ interest group)

While the FMD will likely help combat falsified medicines, many interviewees believed that the focus of the FMD was too strongly on the regulated supply chain:

“Without seeing the real numbers [of falsified medicines], and addressing the real problems, you could say that policymakers chose to overreact, producing a proposal for a directive which has long-term implications for all distributors in the supply chain. […] They are pragmatic and logical, and certainly would add a level of security to the supply chain, but ultimately you have to look really down into where the problems are, that is, the unregulated channels. This does practically nothing. Yeah, something but practically nothing to address the unregulated channel.” (Representative of wholesalers’ interest group)

Based on the results, it would appear that the FMD measures will be costly and will likely influence the future price of medicines. Hinting that the estimated 200 million Euro annual cost may be passed on to the public, a representative of generic manufacturers stated:

“I told my government that we will have to adjust our prices once this is in place, and they said ‘Oh, how much is this going to be?’” (Representative of generic manufacturers’ interest group)

Choosing and promoting the right frame is an effective tool for lobbyists, in that it—if successful—shapes policies to address the promoted frame and thereby affect the legislate outcome [24]. Our results suggest that policymakers rejected early proposals from stakeholders to increase requirements when these were presented as policies to benefit business. However, when the same proposals were presented as a way of protecting patients from falsified medicines, policymakers were more willing—or perhaps even felt obligated—to act.

To outsiders, it appears that the FMD reflects risk aversion among policymakers, proposing a burdensome directive that increases the requirements of an already heavily regulated supply chain and forwards the costs to patients [25,26]. However, as shown in our results, to insiders, the FMD is a tool for establishing barriers to competition and implementing harmonized IT systems capable of generating massive amounts of consumer data.

We have not been able to determine to what degree these different frames were apparent to policymakers. Although patient safety has legitimized the FMD to outsiders, we do not know if patient safety was the dominant frame used in discussions among policymakers. In order to investigate this avenue, we would at the very least have had to have had access to the policymakers who were involved in writing the FMD proposal.

The interest groups we interviewed for this study included both proponents and opponents of the FMD, and all had a clear interest in the outcome of the FMD. There is a risk that we have ignored influential stakeholders outside of the immediate spotlight and only focused on the stakeholders willing to take credit. However, interviewees supported our original approach by not highlighting the influence of stakeholders outside the interest groups we approached.

While at the time of the interviews, work was still being done on the FMD, the events described in this article took place more than seven years ago. Although our interviewees were close to the process of making the FMD, it is likely that our data suffers to some degree from retrospective bias, and we would be naive to assume that recollections of the events are complete. As a result, although this study touches upon some of the major issues with the FMD, it may have ignored potentially important details. However, we have found no major discrepancies between interviewees' recollections.

While our results fit Kingdon’s multiple streams framework, future research may benefit from the use of other approaches, such as frame analysis, to investigate potential issues of corporate bias or risk aversion among policymakers. In order to reject or prove any notion of capture of policymakers by companies, the effects of the FMD for both patients and companies should be investigated, in order to assess if the FMD benefited public or special interest [27].

## 5. Conclusions

Based on a combination of interviews with key actors and analysis of relevant documents, this study attempted to answer the question: what made policymakers prioritize the problem of falsified medicines? Using Kingdon’s multiple streams framework, we show that the problem was highlighted and pushed towards the political agenda by pharmaceutical supply chain actors.

Our results show that brand owners and API manufacturers actively promoted falsified medicines as a problem needing a legislative solution. They framed the issue in a way that would benefit their business interests by focusing on the regulated supply chain rather than on internet sales. When the problem was recognized by policymakers, all interviewed supply chain actors saw this as an opportunity to lobby for policy proposals in line with their business objectives rather than the overall objective of the directive to benefit public health.
